# A Phase 1 Trial of Fimepinostat in Children and Adolescents With Relapsed and Refractory Solid and CNS Tumors

**DOI:** 10.1002/cam4.71417

**Published:** 2025-11-27

**Authors:** David S. Shulman, Kieuhoa T. Vo, Frank M. Balis, Holly Lindsay, Rochelle Bagatell, Andrew E. Place, Susan N. Chi, Suzanne Shusterman, Suzanne Ezrre, Jeffrey Czaplinski, Ketki Bhushan, Pei‐Chi Kao, Wendy B. London, Steven G. DuBois

**Affiliations:** ^1^ Department of Pediatrics, Dana‐Farber/Boston Children's Cancer and Blood Disorders Center Harvard Medical School Boston Massachusetts USA; ^2^ Department of Pediatrics San Francisco School of Medicine, UCSF Benioff Children's Hospital, University of California San Francisco California USA; ^3^ Department of Pediatrics The Children's Hospital of Philadelphia Philadelphia Pennsylvania USA; ^4^ Center for Cancer and Blood Disorders Children's Hospital Colorado Aurora Colorado USA; ^5^ Department of Pediatrics University of Colorado Anschutz Medical Campus Aurora Colorado USA

**Keywords:** CNS tumors, CUDC‐907, dose‐limiting toxicity, fimepinostat, HDAC inhibitor, pediatric oncology, pharmacokinetics, phase 1 trial, PI3K inhibitor, relapsed tumors

## Abstract

**Background:**

Fimepinostat, an oral dual inhibitor of histone deacetylase (HDAC) and phosphatidylinositol‐4,5‐bisphosphate 3‐kinase (PI3K), has shown activity in preclinical models of Myc‐driven pediatric malignancies. This Phase 1 trial aimed to determine the recommended pediatric Phase 2 dose (RPP2D), describe the toxicity profile, and evaluate the pharmacokinetics of fimepinostat in children with relapsed and refractory solid and central nervous system (CNS) tumors.

**Methods:**

This multicenter, Phase 1 study enrolled patients ages 1–21 years with relapsed or refractory solid tumors, CNS tumors, or lymphoma. The dose‐escalation phase followed a 3 + 3 design, starting at 27.5 mg/m^2^ and escalating to 45 mg/m^2^. Following dose escalation, three expansion cohorts were opened including cohorts for patients with Myc(n)‐driven neuroblastoma, Myc‐driven extracranial solid tumors, and diffuse large B‐cell lymphoma or Burkitt lymphoma. The pharmacokinetics of fimepinostat and its metabolites were studied after the first dose.

**Results:**

Twenty‐six patients were enrolled, with 25 receiving treatment. The median age was 13.6 years (range: 4.1–20.9). In the dose‐escalation phase, 12 patients were evaluable for DLT assessment. The RPP2D was initially determined to be 45 mg/m^2^ but was revised to 35 mg/m^2^ after observing DLTs in the dose‐expansion phase. Treatment‐related adverse events were primarily hematologic and gastrointestinal. No objective responses were observed in 23 evaluable patients. Three patients had stable disease for over four cycles, including a patient with *MYCN* amplified neuroblastoma with stable disease for 24 cycles. Pharmacokinetic analysis showed significant interpatient variability and rapid conversion of fimepinostat to its metabolites.

**Conclusion:**

Fimepinostat was tolerable at a dose of 35 mg/m^2^ in children with relapsed and refractory solid and CNS tumors, but lacked significant clinical activity. Discovery of drugs to target Myc continues to be a high priority for childhood cancers.

**Trial Registration:**

ClinicalTrials.gov identifier: NCT02909777

## 1 Introduction

The phosphatidylinositol‐4,5‐bisphosphate 3‐kinase (PI3K) pathway and the family of histone deacetylases (HDAC) are each implicated in the tumorigenesis of multiple childhood cancers. PI3K was identified as a genomic vulnerability in multiple preclinical models of neuroblastoma, medulloblastoma, glioma, and sarcomas [1, 2, 3, 4, 5, 6, 7]. Similarly, HDAC inhibitors have been extensively studied in pediatric malignancies, demonstrating activity in multiple preclinical models, including Myc‐driven tumors [8, 9, 10, 11]. PI3K and HDAC inhibitors have demonstrated modest clinical activity in a range of pediatric solid tumors alone and in combination with other agents [12, 13, 14, 15, 16, 17].

Fimepinostat (formerly known as CUDC‐907) is a small molecule dual inhibitor of HDAC and PI3K. It is available in an oral formulation, including mini‐tabs that can be mixed in soft foods for young children. In a Phase 1 trial of fimepinostat in adults with relapsed/refractory diffuse large B cell and high‐grade B‐cell lymphomas, fimepinostat demonstrated clinical activity in patients with Myc‐altered disease [18]. Supporting this clinical activity in patients with Myc‐driven disease, preclinical work with fimepinostat demonstrated Myc protein downregulation in models of diffuse large B‐cell lymphoma and NUT midline carcinomas and significant anti‐tumor activity [19]. Similarly, fimepinostat was evaluated in preclinical models of *MYCN* amplified neuroblastoma with significant anti‐tumor effects [20]. In the adult clinical trials of fimepinostat, the drug was well tolerated with primarily mild to moderate gastrointestinal and hematologic toxicities, as would be expected with HDAC and PI3K inhibition [18, 21]. To date, dual HDAC and PI3K inhibition has not been evaluated in pediatric patients and prior to this study there were no toxicity data on the use of fimepinostat in children.

The Myc family of transcription factors, which contributes to the pathogenesis of multiple aggressive pediatric extracranial and intracranial solid tumors, has proven challenging to directly inhibit and instead efforts have focused on strategies to downregulate Myc proteins or inhibit their downstream effects [22, 23, 24]. Given our desire to target Myc family proteins and the signal of activity in Myc‐driven adult tumors treated with fimepinostat, we developed a multicenter, open‐label, Phase 1 study of fimepinostat in children and young adults with relapsed or refractory solid tumors, CNS tumors, and lymphoma. The primary objectives of the study were to determine the recommended pediatric Phase 2 dose (RPP2D) of fimepinostat in children with relapsed cancers, to describe the toxicity of fimepinostat and to describe the pharmacokinetics (PKs) of fimepinostat and its metabolites in this population. Secondary objectives included describing the progression‐free survival (PFS), objective response rate and duration of response to fimepinostat in this population. In the published literature, fimepinostat has limited blood–brain barrier penetration (< 10%) [25], and thus patients with CNS tumors of interest were only allowed to enroll during Phase 1.

## 2 Methods

### 2.1 Eligibility

Patients 1–21 years of age were eligible to align with the typical age range of children with relapsed/refractory solid tumors. Patients were required to have a Lansky or Karnofsky performance score > 50% and relapsed/refractory evaluable or measurable disease. For the dose‐escalation phase, participants were required to have histologically confirmed relapsed or refractory solid tumors, CNS tumors, or lymphoma with the only exception being diseases typically diagnosed by clinical imaging such as diffuse intrinsic pontine glioma (DIPG) and optic pathway glioma. Evaluation of fimepinostat in patients with CNS tumors was not a primary aim of our study given reported low blood‐brain penetration; however, there is preclinical evidence of the activity of this agent in patients with pediatric high‐grade glioma and DIPG and thus patients with CNS tumors were allowed to enroll during Phase 1 [25]. During the dose expansion phase, participants were required to have *MYCN* amplified or Myc expressing relapsed/refractory neuroblastoma, diffuse large B‐cell lymphoma or Burkitt lymphoma, or an extracranial solid tumor with *MYC* or *MYCN* amplification or high‐copy gain. At enrollment, patients were required to have absolute neutrophil count (ANC) ≥ 1000 cells/μL, platelet count ≥ 75,000/μL, total bilirubin ≤ 1.5× the upper limit of normal, ALT ≤ 135 U/L and serum albumin ≥ 2 g/dL, adequate age‐related renal function, QTc ≤ 480 ms, < Grade 2 diarrhea and fasting glucose < Grade 2 as defined by the National Cancer Institute Common Toxicity Criteria for Adverse Events version 4 (NCI‐CTCAE v4.0). This trial was approved by the Institutional Review Boards of each enrolling site. Informed consent and assent when appropriate were obtained from each patient prior to enrollment in the study.

### 2.2 Study Design and Treatment

The dose‐escalation portion of this study proceeded according to a 3 + 3 design starting with fimepinostat administered at 27.5 mg/m^2^ orally (78% of the adult equivalent recommended Phase 2 dose) once daily for 5 days followed by two rest days, with treatment repeated each week in 4‐week cycles. Additional dose levels of 18.75, 35, and 45 mg/m^2^ were planned. No intrapatient dose escalation was allowed. Once the RPP2D was determined, three dose‐expansion cohorts were opened at the RPP2D. The study was open at Dana‐Farber/Boston Children's Cancer and Blood Disorders Center (sponsor), UCSF Benioff Children's Hospital, San Francisco, Children's Hospital of Philadelphia, and Texas Children's Hospital.

Fimepinostat was supplied in 10 mg capsules with each capsule containing four 2.5 mg mini‐tabs that could be administered to children unable to swallow pills by mixing the appropriate number of mini‐tabs with food. Fimepinostat was recommended to be administered at approximately the same time each day with food.

### 2.3 Clinical Endpoints

Toxicity grading was performed using CTCAE version 4.0. Hematologic dose‐limiting toxicity (DLT) was defined as Grade 4 thrombocytopenia, Grade 4 anemia, or Grade 4 neutropenia of any duration in the absence of bone marrow disease progression seen on a clinically indicated bone marrow biopsy. Grade 3 thrombocytopenia in association with Grade 2 or higher bleeding, or delay in the start of a subsequent cycle by > 14 days due to thrombocytopenia or neutropenia in the absence of bone marrow disease progression seen on a clinically indicated bone marrow biopsy was also considered to be hematologic DLT. Non‐hematologic DLT was defined as any Grade 3 or higher non‐hematologic toxicity attributable to fimepinostat with the exclusion of: Grade 3 nausea and vomiting, anorexia or dehydration resolving to < Grade 2 within 72 h; Grade 3 AST or ALT liver enzyme elevation that returns to < Grade 2 within 4 days; Grade 4 febrile neutropenia in the absence of clinical or laboratory documentation of infection; Grade 3 hypophosphatemia, hypokalemia, hypocalcemia, or hypomagnesemia that responds to oral supplementation. Grade 2 non‐hematologic toxicities persisting for ≥ 7 days or that were significant enough to cause a treatment interruption were also considered a DLT. Any non‐hematologic toxicity causing > 14‐day delay in the start of a treatment cycle was considered a DLT.

For patients with non‐neuroblastoma and non‐CNS tumors, response was assessed by the Response Evaluation Criteria in Solid Tumors (RECIST) version 1.1 [26]. For patients with neuroblastoma, the New Approaches to Neuroblastoma Therapy (NANT) response criteria version 1.2 were used [27]. For patients with CNS tumors, the RANO criteria were used [28]. Disease evaluations were performed at baseline, following cycles, 2, 4, 6 and then every third cycle. PFS time was defined as the time from study enrollment until the first occurrence of an event (relapse, progression, or death from any cause), or until last contact if no event occurred.

### 2.4 Pharmacokinetic Studies

All patients had PK sampling performed after the first dose. Blood samples were collected on Day 1 pre‐dose, at 9 time points over the subsequent 24 h, and on Day 15 pre‐dose. Additional pre‐dose samples were collected prior to the initiation of Cycles 3 and 4 for patients remaining on study. PK samples were analyzed for fimepinostat and its two metabolites using high‐performance liquid chromatography with tandem mass spectrometry. Pharmacokinetic parameters for fimepinostat and its 2 metabolites were derived using non‐compartmental methods with the Phoenix NCA module (Certara, Princeton, NJ).

### 2.5 Statistical Analysis

Patients were evaluable for the dose finding objective if they had received ≥ 75% of the total planned dose of fimepinostat in Cycle 1 and were followed until Day 28 of the first cycle, or if they had a DLT in Cycle 1, regardless of the total fimepinostat dose received. The primary endpoint for dose escalation was DLT attributable to fimepinostat in the first cycle (binary, DLT or no DLT). To be evaluable for the analysis of response, patients must have received at least one dose of fimepinostat and have undergone at least one response assessment. To be evaluable for the analysis of PFS, patients must have received at least one dose of fimepinostat.

The primary endpoint for the dose expansion portion of the study was best overall objective response, with a responder defined as having a partial or complete response. An exact single‐stage Phase 2 design was utilized to test the null hypothesis of a response rate of ≤ 1% against an alternative response rate of ≥ 21%, separately within each of the three cohorts, with 10 planned patients per cohort. Within each cohort, if one or more responders were observed, we were able to reject the null hypothesis with a type 1 error of 0.096 and power of 90%. A pre‐defined toxicity monitoring rule was utilized during dose expansion whereby if ≥ 2 out of 6 or ≥ 3 out of 9 patients experienced a DLT, a formal safety review would be triggered. Exact Clopper–Pearson 95% confidence intervals were placed on the toxicity rate and the response rate, calculated within evaluable patient subgroups. Kaplan–Meier methods were used to estimate PFS. No inferential testing was performed. SAS version 9.4 was used for analysis.

## 3 Results

### 3.1 Patient Characteristics

**FIGURE 1:**
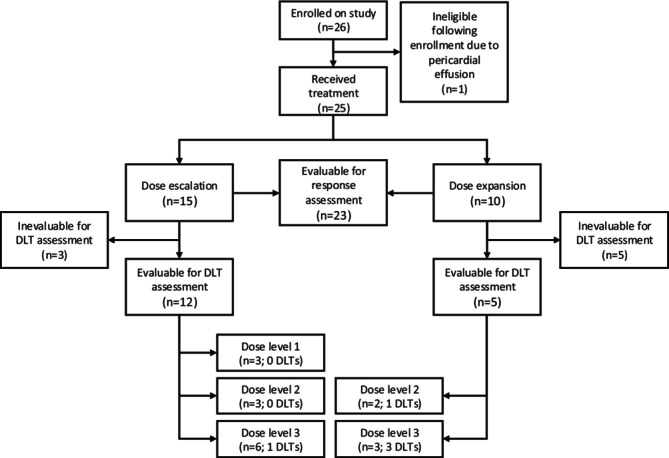
Study consort diagram.

Between 9/7/2016 and 1/20/2023, 26 patients enrolled in the study and one patient was subsequently determined to be ineligible prior to the initiation of therapy. The median age was 13.6 years (range: 4.1–20.9) and 48% of patients were male (Table 1). Fifteen patients were enrolled in the dose escalation phase of the study, including one patient with *Myc* amplified osteosarcoma. Six patients were enrolled in the Myc‐driven extracranial solid tumor expansion cohort, and four were enrolled in the Myc‐driven neuroblastoma expansion cohort (Figure 1), for a total of 11 patients with Myc‐driven disease. The study closed prior to completion of enrollment in the dose expansion phase due to discontinuation of development of the study agent and slow accrual. Enrollments by study site are shown in Table S1.

**TABLE 1:** Patient and disease characteristics (*n* = 25).

	Overall (*n* = 25)
	**Median (range)**
Age at diagnosis (years)	13.6 (4.1–20.9)
Number of prior lines of therapy	5 (1–12)
	***n* (%)**
Sex	
Male	12 (48%)
Female	13 (52%)
Race	
White	15 (60%)
Non‐white	10 (40%)
Ethnicity	
Hispanic	3/22 (14%)
Non‐Hispanic	19/22 (86%)
Unknown	3
Histology	
Osteosarcoma	8 (32%)
Neuroblastoma	4 (16%)
Alveolar rhabdomyosarcoma	4 (16%)
Ependymoma	2 (8%)
Ewing sarcoma	2 (8%)
Diffuse intrinsic pontine glioma	2 (8%)
CNS non‐germinomatous germ cell tumor	1 (4%)
Myxoid liposarcoma	1 (4%)
Alveolar soft part sarcoma	1 (4%)

### 3.2 Dose Limiting Toxicities and Recommended Phase 2 Dose

Of the 15 patients who enrolled during dose escalation, 12 were evaluable for Cycle 1 DLT assessment. There were no DLTs among the three patients treated at dose level 1 (27.5 mg/m^2^), no DLTs in three patients treated at dose level 2 (35 mg/m^2^) and one DLT in six patients treated at dose level 3 (45 mg/m^2^). Following the dose escalation phase, dose level 3, 45 mg/m^2^ on a 5‐day on, 2‐day off continuous schedule, was declared the RPP2D.

Among the first three patients who enrolled in the dose expansion cohorts at the 45 mg/m^2^ dose level, three had Cycle 1 DLTs. This triggered pre‐specified safety stopping rules and toxicity was re‐evaluated. The recommended Phase 2 dose was revised to dose level 2, 35 mg/m^2^ 5‐day on, 2‐day off. Following the revision of the recommended Phase 2 dose, an additional seven patients were enrolled in the expansion cohorts at dose level 2. Among those seven patients, two met criteria to be fully evaluable for dose limiting toxicity assessment and one had a Cycle 1 DLT. In total, 10 patients enrolled in dose level 2 and five were evaluable for Cycle 1 DLT assessment. Thus, the observed DLT rate at dose level 2 was 20% (95% CI: 0.5%–72%). Cycle 1 DLTs included, Grade 3 diarrhea, Grade 4 neutropenia, Grade 4 thrombocytopenia, Grade 3 acidosis, and Grade 3 headache. A summary of all DLTs is shown in Table S2.

### 3.3 Other Toxicity

In Table 2, we report Grades 1 and 2 treatment‐related AEs that occurred in greater than 10% of patients and all Grade 3 and higher AEs. The most common treatment‐related AEs of any grade were platelet count decreased (*n* = 20), lymphocyte count decreased (*n* = 19), anemia (*n* = 17), diarrhea (*n* = 17), white blood cell decreased (*n* = 17), nausea (*n* = 16), fatigue (*n* = 15), and neutrophil count decreased (*n* = 15). Grade 1 hyperglycemia was reported in 32% of patients, with no higher grade hyperglycemia observed. Grade 3 or higher non‐hematologic AEs occurred in 4 (16%) patients. Grade 3 or higher hematologic AEs occurred in 16 (64%) patients. Treatment‐related AEs led to dose reductions in four patients and included Grade 3 acidosis, Grade 3 headache, Grade 3 diarrhea, and Grade 4 neutrophil count decreased. One patient discontinued treatment due to toxicity. Eight patients died during the study and none of the deaths were due to causes related to study treatment.

**TABLE 2:** Treatment‐associated adverse events including any Grade 1/2 adverse event affecting > 10% of patients and all Grade 3 or higher adverse events.^a^

Toxicity category CTCAE v4.0	Toxicity description CTCAE v4.0	Grade 1	Grade 2	Grade 3	Grade 4	Any grade
		*N* (%)	*N* (%)	*N* (%)	*N* (%)	*N* (%)
Blood and lymphatic system disorders	Anemia	10 (40%)	4 (16%)	3 (12%)	0 (0%)	17 (68%)
Gastrointestinal disorders	Abdominal pain	6 (24%)	0 (0%)	0 (0%)	0 (0%)	6 (24%)
	Diarrhea	13 (52%)	3 (12%)	1 (4%)	0 (0%)	17 (68%)
	Nausea	14 (56%)	2 (8%)	0 (0%)	0 (0%)	16 (64%)
	Vomiting	14 (56%)	0 (0%)	0 (0%)	0 (0%)	14 (56%)
General disorders and admin site conditions	Fatigue	8 (32%)	7 (28%)	0 (0%)	0 (0%)	15 (60%)
Investigations	Alanine aminotransferase increased	4 (16%)	0 (0%)	1 (4%)	0 (0%)	5 (20%)
	Aspartate aminotransferase increased	6 (24%)	0 (0%)	0 (0%)	0 (0%)	6 (24%)
	Blood bilirubin increased	3 (12%)	0 (0%)	0 (0%)	0 (0%)	3 (12%)
	Creatinine increased	3 (12%)	0 (0%)	0 (0%)	0 (0%)	3 (12%)
	Lymphocyte count decreased	5 (20%)	2 (8%)	6 (24%)	3 (12%)	16 (64%)
	Neutrophil count decreased	5 (20%)	2 (8%)	4 (16%)	2 (8%)	13 (52%)
	Platelet count decreased	11 (44%)	4 (16%)	4 (16%)	1 (4%)	20 (80%)
	Weight loss	1 (4%)	3 (12%)	0 (0%)	0 (0%)	4 (16%)
	White blood cell decreased	11 (44%)	5 (20%)	0 (0%)	1 (4%)	17 (68%)
Metabolism and nutrition disorders	Acidosis	0 (0%)	0 (0%)	1 (4%)	0 (0%)	1 (4%)
	Anorexia	7 (28%)	6 (24%)	0 (0%)	0 (0%)	13 (52%)
	Dehydration	0 (0%)	3 (12%)	0 (0%)	0 (0%)	3 (12%)
	Hyperglycemia	8 (32%)	0 (0%)	0 (0%)	0 (0%)	8 (32%)
	Hyperkalemia	4 (16%)	0 (0%)	0 (0%)	0 (0%)	4 (16%)
	Hypermagnesemia	3 (12%)	0 (0%)	0 (0%)	0 (0%)	3 (12%)
	Hypoalbuminemia	2 (8%)	1 (4%)	0 (0%)	0 (0%)	3 (12%)
	Hypokalemia	5 (20%)	0 (0%)	0 (0%)	1 (4%)	6 (24%)
	Hyponatremia	5 (20%)	0 (0%)	0 (0%)	0 (0%)	5 (20%)
	Hypophosphatemia	2 (8%)	0 (0%)	1 (4%)	0 (0%)	3 (12%)
Musculoskeletal and connective tissue disorders	Myalgia	3 (12%)	0 (0%)	0 (0%)	0 (0%)	3 (12%)
Nervous system disorders	Headache	2 (8%)	3 (12%)	1 (4%)	0 (0%)	6 (24%)
	Hypersomnia	3 (12%)	1 (4%)	0 (0%)	0 (0%)	4 (16%)
Vascular disorders	Hypertension	4 (16%)	0 (0%)	0 (0%)	0 (0%)	4 (16%)

^a^ No Grade 5 adverse events were observed.

### 3.4 Efficacy

**FIGURE 2:**
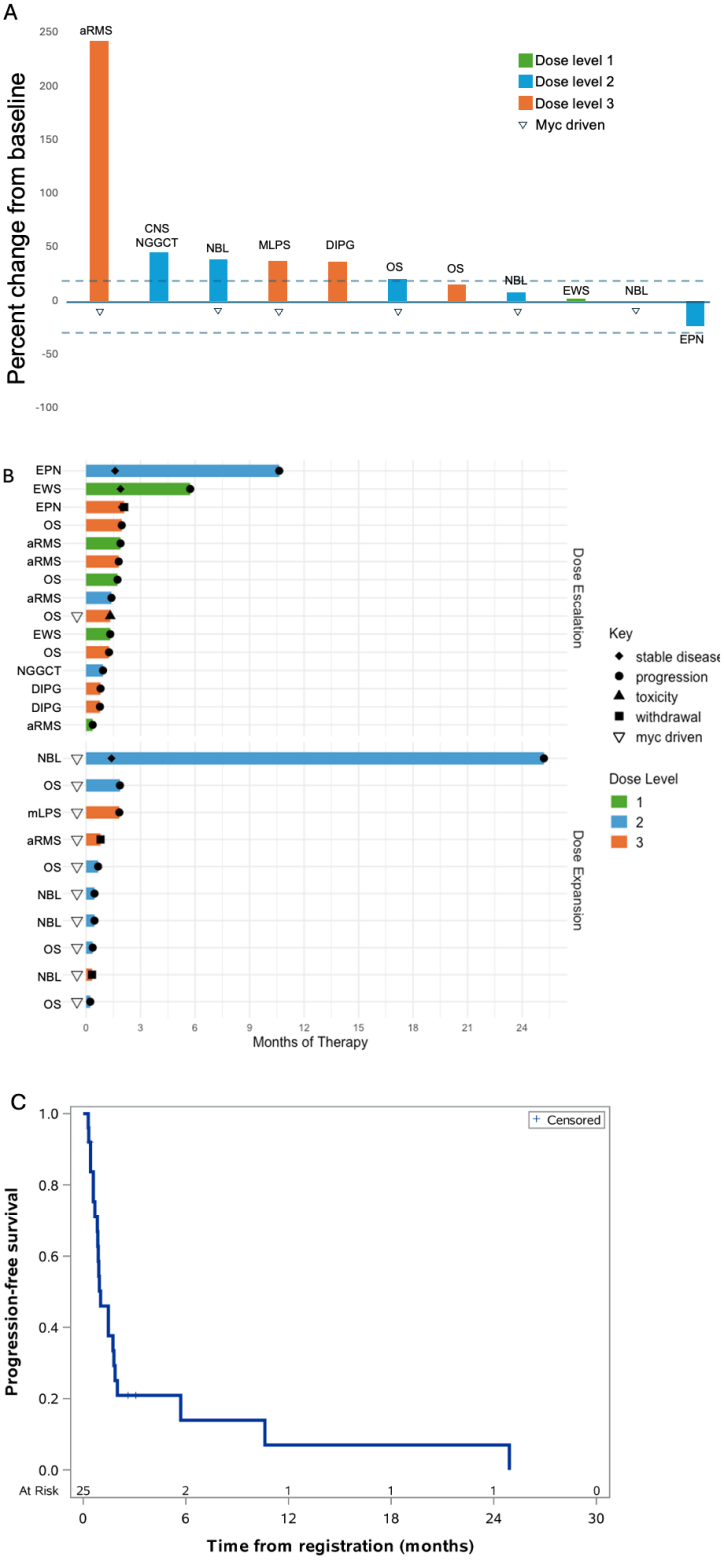
(A) Waterfall plot of the maximum decrease from baseline in tumor size for patients with measurable disease (*n* = 11 patients with measurable disease and at least one follow‐up disease assessment). (B) Swimmer plot of all patients from the dose escalation and dose expansion portions of the study (*n* = 25). (C) Kaplan–Meier curve of progression‐free survival (*n* = 25). aRMS, alveolar rhabdomyosarcoma; DIPG, diffuse intrinsic pontine glioma; EPN. ependymoma; EWS, Ewing sarcoma; mLPS, myxoid liposarcoma; NBL, neuroblastoma; NGGCT, CNS non‐germinomatous germ cell tumor; OS, osteosarcoma.

There were 23 patients evaluable for response assessment in the dose escalation and dose expansion arms of the study. There were no patients with a RECIST‐defined partial or complete response. There were three patients with stable disease for 4 cycles or greater. Patients with the longest duration of disease control were a patient with Ewing sarcoma (5.6 months), a patient with ependymoma (10.5 months), and a patient with *MYCN* amplified neuroblastoma (24.8 months). In Figure 2A, we show best response by dose level and disease histology for the 10 patients who had measurable disease at baseline and at least one completed follow‐up disease assessment. In Figure 2B, we show a swimmer plot of duration on study therapy. The 6‐month and 1‐year estimated PFS rates were 14% ± 7.9% and 7% ± 6.3%, respectively (Figure 2C).

### 3.5 Pharmacokinetics

**FIGURE 3:**
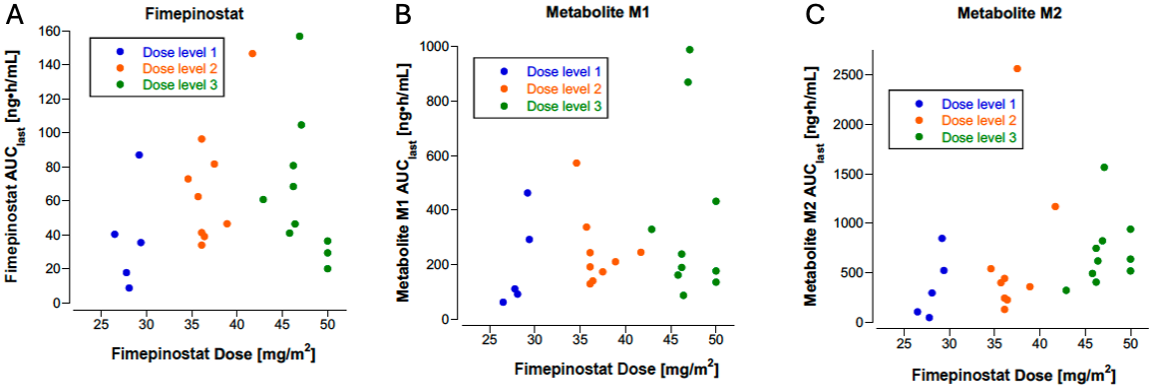
Fimepinostat pharmacokinetic parameters: AUC_last_ by dose level (*n* = 24) for drug (A), metabolite M1 (B), and metabolite M2 (C).

The PK parameters of fimepinostat and two metabolites (M1, inactive and M2, limited PI3K inhibitor activity) are listed in Table 3. There was significant interpatient variability, as reported in the adult Phase 1 study of fimepinostat (Figure S1) [21]. The area under the concentration‐time curve to the last measured timepoint (AUC_last_) generally increased by dose level, with the most notable increase between dose level 1 and dose level 2 (Figure 3A). The parent drug was rapidly cleared, but there was accumulation of the M1 and M2 metabolites at dose levels 2 and 3 (Figure 3B,C). Pre‐cycle troughs prior to Cycles 3 and 4 showed ongoing accumulation of the M1 and M2 metabolites and clearance of the parent drug.

**TABLE 3:** PK parameters for fimepinostat and its metabolites.

			Parameter				
			*C*_max_ (ng/mL) *n* = 5^a^	AUC_last_ (h*ng/mL) *n* = 5	C1D15 trough *n* = 3	C3D1 trough *n* = 1	C4D1 trough *n* = 1
Dose level 1	Fimepinostat	Median	6.92	35.55	0	0	0
		Min	4.32	8.87	0	0	0
		Max	32.93	86.98	0	0	0
	Metabolite M1^b^	Median	8.36	110.72	18.8	0	0
		Min	6.44	61.64	0	0	0
		Max	28.81	462.83	26.28	0	0
	Metabolite M2^c^	Median	24.15	297.01	22.47	0	0
		Min	2.93	48.48	0	0	0
		Max	56.52	848.91	42.32	0	0
			***n* = 9**	***n* = 9**	***n* = 7**	***n* = 3**	***n* = 2**
Dose level 2	Fimepinostat	Median	25.99	62.55	0	0	0
		Min	9.61	34.08	0	0	0
		Max	109.65	146.55	0	0	0
	Metabolite M1	Median	14.63	210.45	15.67	10.99	4.8
		Min	9.36	128.71	0	0	0
		Max	37.43	572.52	27.95	16.46	9.59
	Metabolite M2	Median	26.47	400.35	14.12	3.81	15.79
		Min	10.39	131.58	0	0	0
		Max	210.99	2561.27	47.05	4.50	31.58
			***n* = 10**	***n* = 10**	***n* = 8**	***n* = 0**	***n* = 0**
Dose level 3	Fimepinostat	Median	16.51	53.65	0	n/a	n/a
		Min	6.81	20.19	0	n/a	n/a
		Max	41.48	156.69	3.11	n/a	n/a
	Metabolite M1	Median	14.90	213.74	5.24	n/a	n/a
		Min	5.03	86.71	0	n/a	n/a
		Max	68.66	988.41	23.09	n/a	n/a
	Metabolite M2	Median	49.58	629.37	2.16	n/a	n/a
		Min	24.26	323.75	0	n/a	n/a
		Max	153.71	1565.48	43.45	n/a	n/a

^a^ Sample size shown indicates the number of patients with available PK data at each dose level and each timepoint.

^b^ The M1 metabolite is inactive.

^c^ The M2 metabolite has minimal PI3K inhibitory activity.

## 4 Discussion

The primary objectives of this trial were to determine the RPP2D of fimepinostat, describe the toxicities of this agent and describe the PKs in children with relapsed solid tumors, CNS tumors and lymphoma. The RPP2D of fimepinostat was 35 mg/m^2^ given in a 5‐day on, 2‐day off schedule of a 28‐day cycle. The study was terminated prior to completing the pre‐specified 3 + 3 dose finding rules with five patients treated at 35 mg/m^2^ who were evaluable for toxicity assessment. The observed toxicities were primarily hematologic and gastrointestinal. The PK parameters demonstrated interpatient variability similar to that seen in adult trials of fimepinostat. Although three patients remained on therapy for greater than 4 months, there were no objective responses seen in patients with multiple cancers of interest. Thus, there are insufficient data to suggest further study of this agent as monotherapy in this patient population.

In the dose escalation portion of this study, three patients each were treated at dose levels 1 and 2 without DLTs. At dose level 3, six patients were treated with one DLT, and thus dose level 3 was initially deemed the RPP2D. In dose expansion, the first three patients experienced DLTs, yielding 4/9 patients with DLTs at 45 mg/m^2^, exceeding the pre‐specified toxicity threshold. The RPP2D was revised from 45 mg/m^2^ to dose level 2, 35 mg/m^2^. Seven patients were subsequently treated at dose level 2 during the dose expansion phase of the trial, but only two of those patients were evaluable for DLT assessment mainly due to early progression. This highlights the aggressive nature of Myc‐driven pediatric cancers. One of those two patients had a Cycle 1 DLT. Thus, the observed DLT rate was 20% with a wide confidence interval. With the available data, we recommend 35 mg/m^2^ as the RPP2D, acknowledging that optimally there should have been at least six patients evaluable for toxicity assessment treated at dose level 2. Fimepinostat has been most extensively studied in adults with lymphoma and the established adult fixed dose is 60 mg on a 5‐day on, 2‐day off schedule [18, 21, 29], which is similar to the BSA‐based dose level we recommend in children.

We observed wide intrapatient variability in fimepinostat exposure (AUC). This is an expected result for an oral small molecule inhibitor with rapid clearance and was analogous to what was seen in the adult Phase 1 study of fimepinostat [21]. We observed rapid clearance of the parent drug, with accumulation of the two metabolites (M1, M2). The AUC generally increased by dose level but with considerable overlap across the three dose levels. Toxicity was also dose dependent, with a 44% DLT rate at dose level 3. Hematologic AEs were common with thrombocytopenia, lymphopenia, neutropenia and anemia observed in most patients. Non‐hematologic toxicities were generally mild. This patient population was heavily pre‐treated, with a median of 5 prior lines of therapy, potentially contributing to the observed hematologic toxicities.

There were three patients on this study with prolonged stable disease, including one patient with *MYCN‐amplified* neuroblastoma. The duration of disease control (24 months) is distinctly unusual for patients with relapsed *MYCN‐amplified* neuroblastoma [30]. The PK data for this patient are shown in Figure S1 case 24, and align with the PK data we observed in other patients treated at dose level 2. All three additional patients with Myc‐driven neuroblastoma had early progression. No patients had an objective response. Fimepinostat has previously demonstrated clinical activity in Myc‐driven lymphomas [18, 21, 29]. In addition, multiple lines of evidence demonstrated preclinical activity of fimepinostat in Myc‐driven pediatric tumors, including neuroblastoma [19, 20]. In our study, there were 11 patients treated with Myc‐driven tumors, all of whom were treated at dose levels 2 or 3, and none with observed responses. Due to low accrual and discontinuation of development of this agent, the dose expansion cohorts did not fully accrue; however given the lack of responses seen across 23 patients with response‐evaluable disease, we concluded that this agent does not warrant further evaluation as monotherapy in this patient population.

While our study was open at four centers from 9/7/2016 to 1/20/2023, the study did not complete accrual. There were only five patients who were evaluable for DLT assessment treated at dose level 2 leading to decreased confidence in calling this dose level the RPP2D. However, this dose level aligns with the recommended Phase 2 dose in adults, and thus should this agent warrant further study in children, we would recommend moving forward with 35 mg/m^2^ on a 5‐day on, 2‐day off schedule. We have provided PK data of the primary drug and the two dominant metabolites and note that it is possible that metabolite accumulation at the highest dose level may have contributed to the observed toxicity. This study also did not complete accrual to the dose expansion cohorts, limiting our ability to rigorously determine the response rate of this agent in Myc‐driven pediatric tumors. We observed no responses among 23 patients who were evaluable for response assessment, and 11 patients with Myc‐driven tumors. Similarly, evaluation of this drug for treatment of adult Myc‐driven lymphomas was discontinued due to the degree of clinical activity [29]. Finally, assessment of PI3K pathway mutations and HDAC activity was not possible for the patients treated on this trial. While this could have impacted assessment of efficacy, the impact of dual PI3K and HDAC inhibition was thought to occur through inhibition of Myc activity. We also note that PI3K pathway mutations are very rare in pediatric cancers, and thus unlikely to have had a major impact on the outcome of this trial [31].

In conclusion, fimepinostat was tolerable in children with relapsed and refractory solid tumors, with primarily hematologic and gastrointestinal toxicity. We recommend 35 mg/m^2^ as the RPP2D in this patient population. This agent lacked clinical activity to match the available preclinical data. These findings are in line with a recent ACCELERATE multistakeholder pediatric strategy forum which concluded that there is a limited role for PI3K inhibition in most pediatric cancers [31]. Future efforts to target Myc‐driven tumors should focus on combination approaches.

## Author Contributions

D.S.S.: conceptualization, formal analysis, investigation, supervision, visualization, formal analysis, writing – original draft, reviewing editing. K.T.V.: investigation, supervision, writing. F.M.B.: data curation, formal analysis, visualization, writing. H.L.: investigation, supervision, writing. R.B.: investigation, supervision, writing. A.E.P.: investigation, supervision, writing. S.N.C.: investigation, supervision, writing. S.S.: investigation, supervision, writing. S.E.: investigation, data curation, writing. J.C.: data curation, project administration, writing. K.B.: data curation, project administration, writing. P.‐C.K.: data curation, formal analysis, visualization, writing. W.B.L.: conceptualization, data curation, formal analysis, supervision, visualization, writing. S.G.D.: conceptualization, formal analysis, investigation, funding acquisition, project administration, supervision, writing.

## Funding

This work was supported by Dana‐Farber Cancer Institute, The Hyannisport Jimmy Fund Golf Fund; Alex's Lemonade Stand Foundation for Childhood Cancer, Centers of Excellence Grant; Kellen Family Foundation.

## Ethics Statement

This trial was approved by the Dana‐Farber/Harvard Cancer Center Institutional Review Board and the institutional review boards of each participating institution.

## Consent

Informed consent was reviewed and signed by all patients prior to study entry.

## Conflicts of Interest

D.S.S. reports consulting fees from Boehringer Ingelheim. S.G.D. reports consulting fees from Amgen, Bayer, EMD Serono, InhibRx, and Jazz and travel expenses from Loxo, Roche, and Salarius. W.B.L. reports prior consulting fees from Merck Sharp & Dohme Corp, ArQule Inc., and Jubilant DraxImage Inc. for service on Data Safety Monitoring Boards, from Y‐mAbs Therapeutics Inc. for service on their Scientific Advisory Board, and as a consultant to Healthcasts.

## Supporting information


**Figure S1:** Cycle 1, Days 1 and 2 pharmacokinetics for each patient at each dose level for parent drug (CUDC‐907/fimepinostat) and the two primary metabolites. Data are shown in log‐scale.

**Table S1:** Patient enrollment by site.
**Table S2:** Description of dose limiting toxicities in pediatric patients treated with fimepinostat (*n* = 5 patients reported DLTs).

## Data Availability

The data that support the findings of this study are available on request from the corresponding author. The data are not publicly available due to privacy or ethical restrictions.
