# Supplementary material for: A Phase 1 Trial of Fimepinostat in Children and Adolescents With Relapsed and Refractory Solid and CNS Tumors
Source: Cancer Med. 2025 Nov 27;14(23):e71417. doi: 10.1002/cam4.71417 (PMC12659765; doi:10.1002/cam4.71417)
Supplement: Supplementary file 1 — Figure S1: Cycle 1, Days 1 and 2 pharmacokinetics for each patient at each dose level for parent drug (CUDC‐907/fimepinostat) and the two primary metabolites. Data are shown in log‐scale. [file CAM4-14-e71417-s002.pdf]

DOSE LEVEL 1

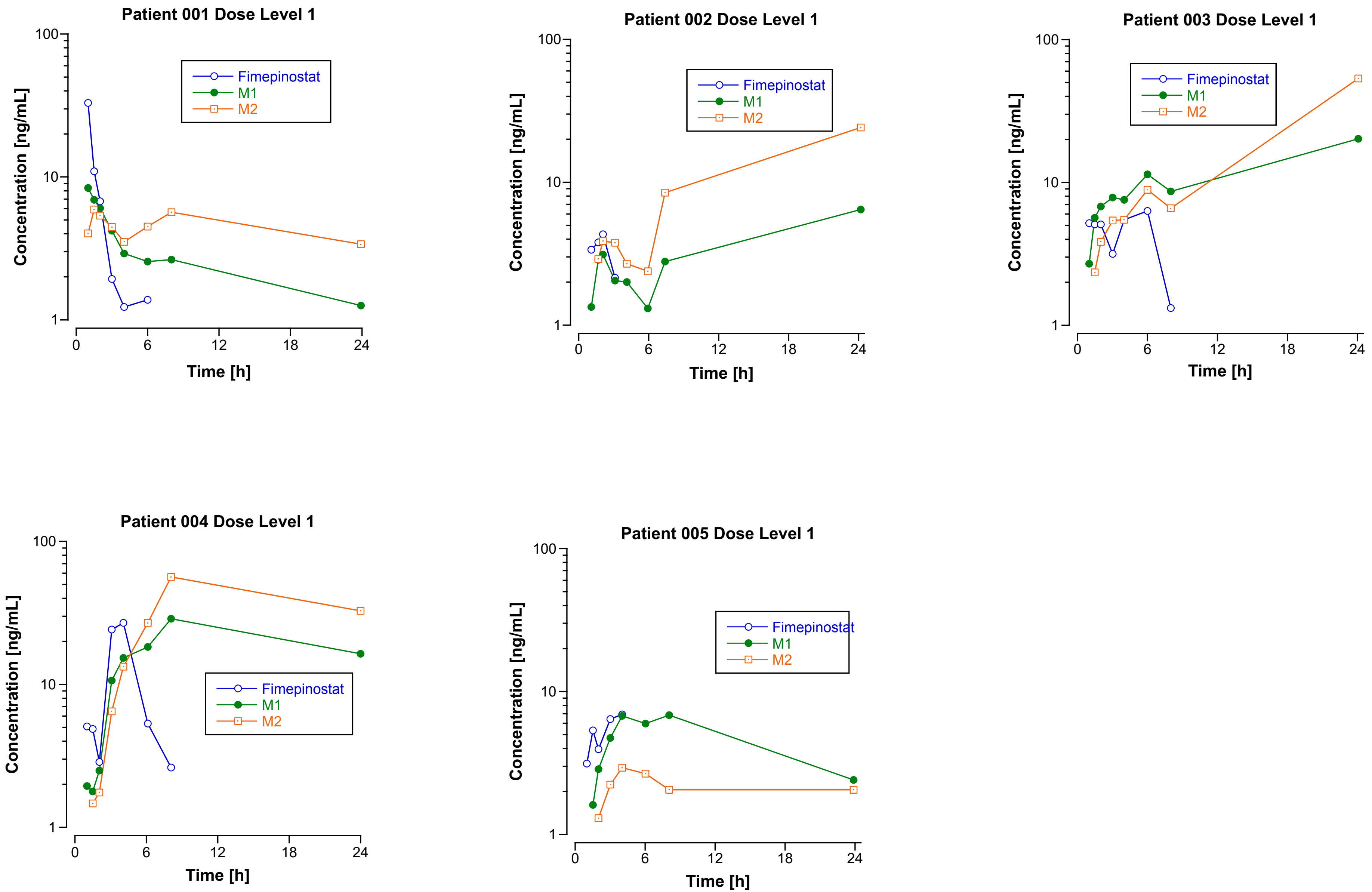

Patient 001 Dose Level 1
Patient 002 Dose Level 1
Patient 003 Dose Level 1
Patient 004 Dose Level 1
Patient 005 Dose Level 1
Concentration [ng/mL]
Time [h]
100
10
1
0
6
12
18
24
Fimepinostat
M1
M2

DOSE LEVEL 2

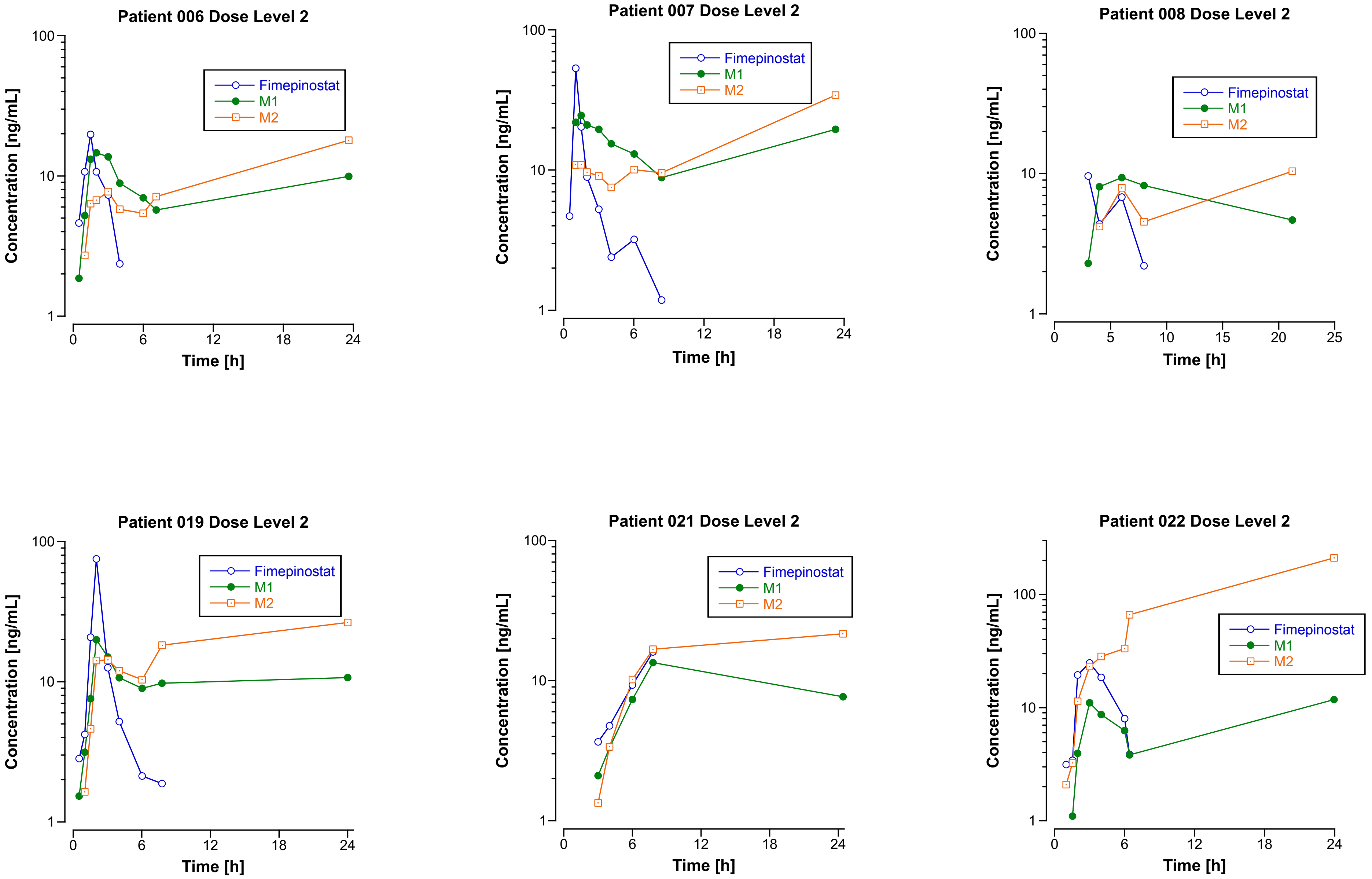

Patient 006 Dose Level 2
Patient 007 Dose Level 2
Patient 008 Dose Level 2
Patient 019 Dose Level 2
Patient 021 Dose Level 2
Patient 022 Dose Level 2
Fimepinostat
M1
M2
Concentration [ng/mL]
Time [h]

DOSE LEVEL 2

Patient 023 Dose Level 2

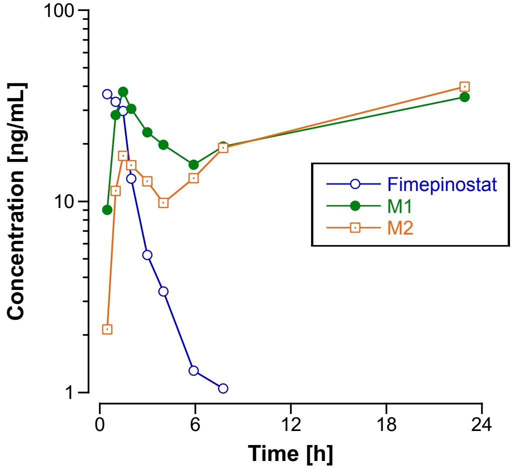

Patient 024 Dose Level 2

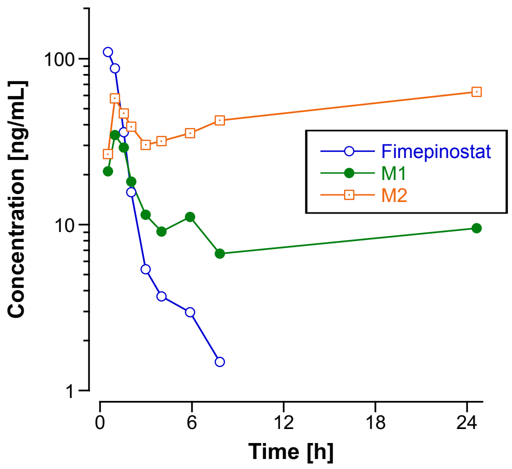

Patient 025 Dose Level 2

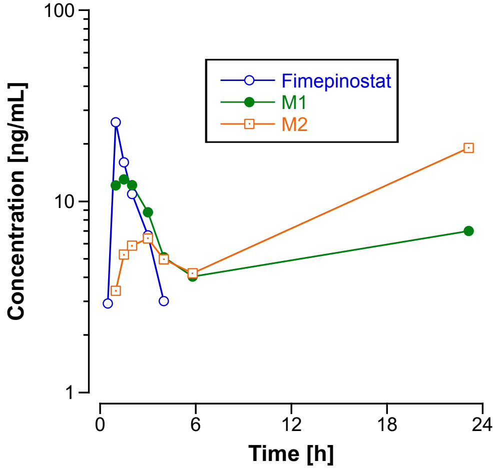

DOSE LEVEL 3

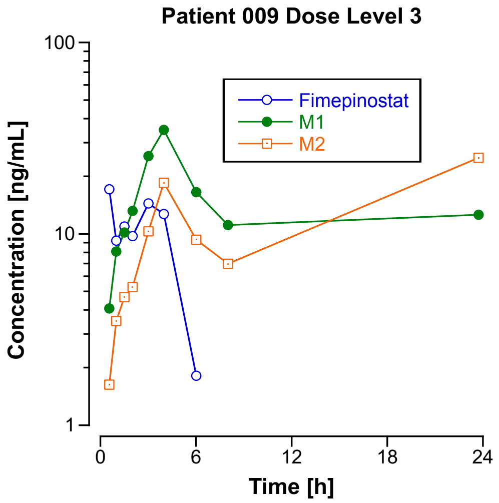

Patient 009 Dose Level 3
Concentration [ng/mL]
100
10
1
0
6
12
18
24
Time [h]
Fimepinostat
M1
M2

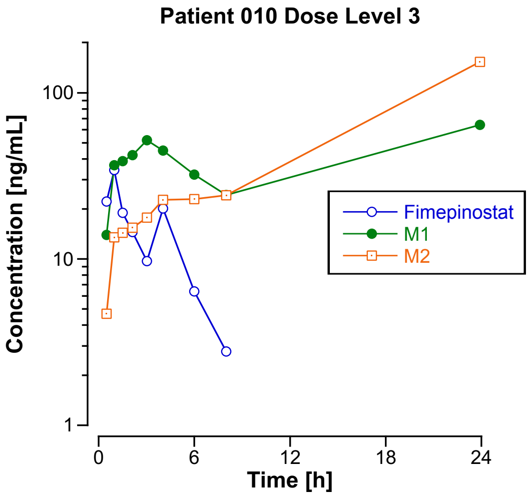

Patient 010 Dose Level 3
Concentration [ng/mL]
100
10
1
0
6
12
18
24
Time [h]
Fimepinostat
M1
M2

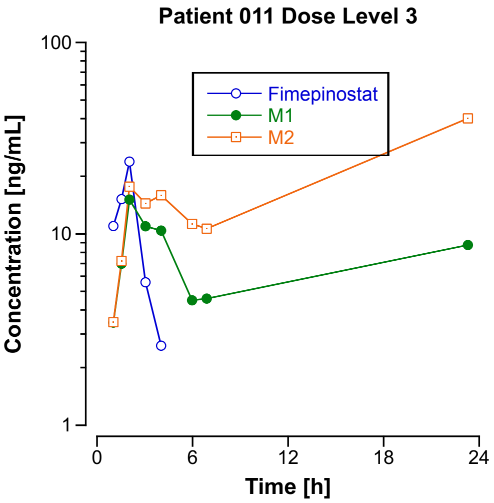

Patient 011 Dose Level 3
Concentration [ng/mL]
100
10
1
0
6
12
18
24
Time [h]
Fimepinostat
M1
M2

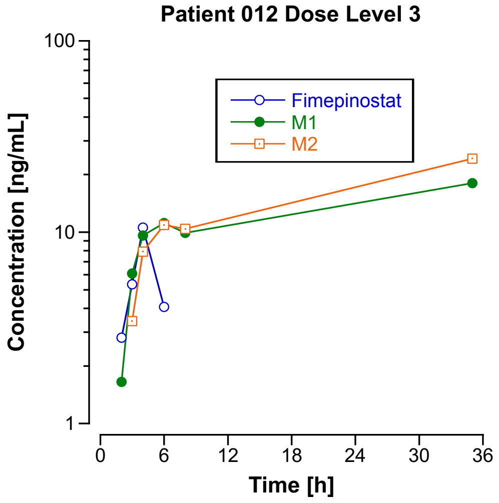

Patient 012 Dose Level 3
Concentration [ng/mL]
100
10
1
0
6
12
18
24
30
36
Time [h]
Fimepinostat
M1
M2

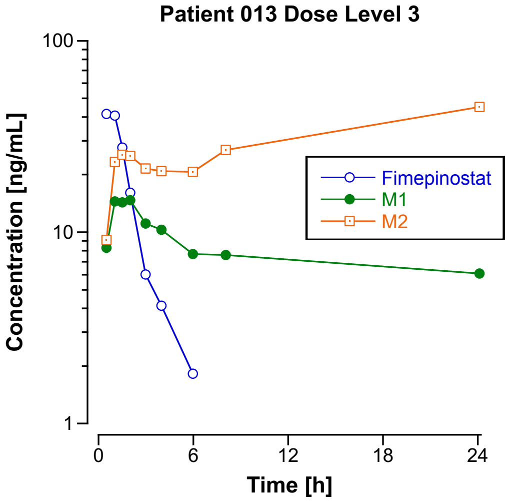

Patient 013 Dose Level 3
Concentration [ng/mL]
100
10
1
0
6
12
18
24
Time [h]
Fimepinostat
M1
M2

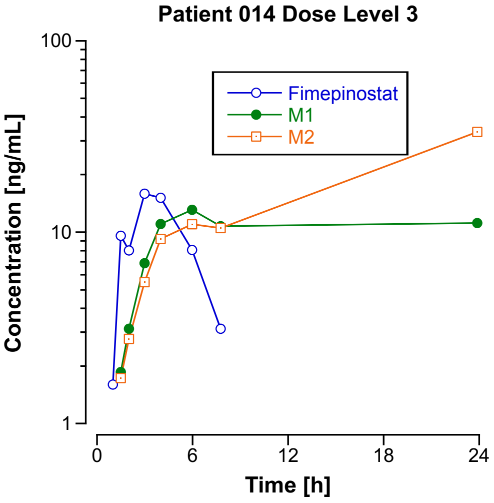

Patient 014 Dose Level 3
Concentration [ng/mL]
100
10
1
0
6
12
18
24
Time [h]
Fimepinostat
M1
M2

DOSE LEVEL 3

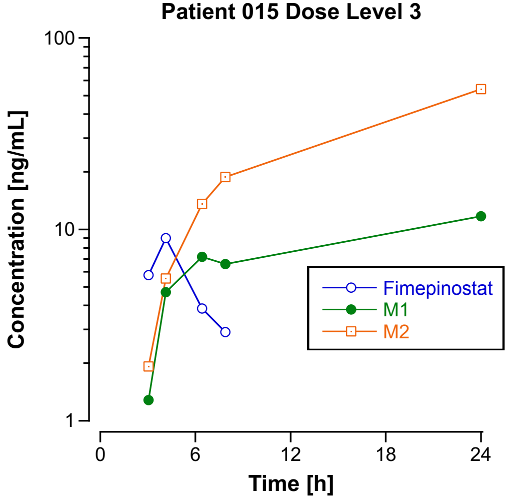

Patient 015 Dose Level 3
Concentration [ng/mL]
100
10
1
0
6
12
18
24
Time [h]
Fimepinostat
M1
M2

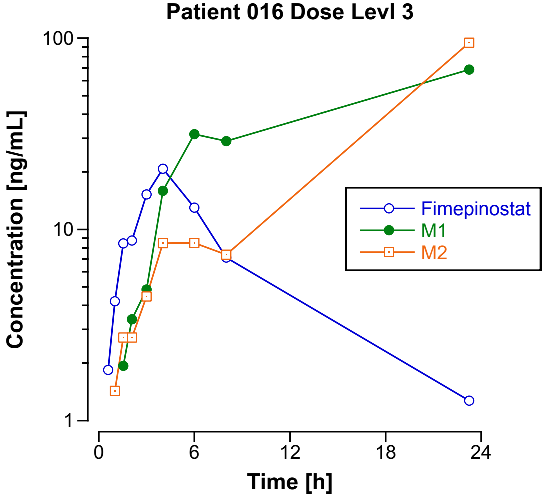

Patient 016 Dose Levl 3
Concentration [ng/mL]
100
10
1
0
6
12
18
24
Time [h]
Fimepinostat
M1
M2

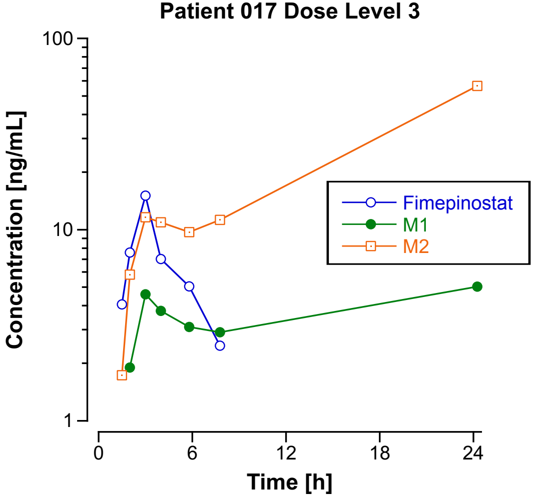

Patient 017 Dose Level 3
Concentration [ng/mL]
100
10
1
0
6
12
18
24
Time [h]
Fimepinostat
M1
M2

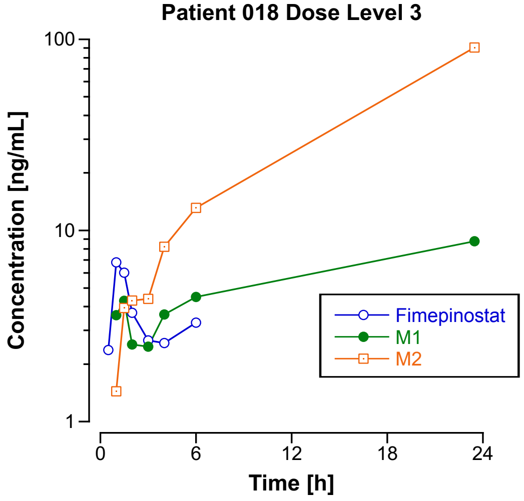

Patient 018 Dose Level 3
Concentration [ng/mL]
100
10
1
0
6
12
18
24
Time [h]
Fimepinostat
M1
M2
