# Supplementary material for: A Phase 1 Trial of Fimepinostat in Children and Adolescents With Relapsed and Refractory Solid and CNS Tumors
Source: Cancer Med. 2025 Nov 27;14(23):e71417. doi: 10.1002/cam4.71417 (PMC12659765; doi:10.1002/cam4.71417)
Supplement: Supplementary file 2 — Table S1: Patient enrollment by site. Table S2: Description of dose limiting toxicities in pediatric patients treated with fimepinostat (n = 5 patients reported DLTs). [file CAM4-14-e71417-s001.docx]

**Supplemental Table 1**: Patient enrollment by site.

| **Site** | **Number of enrollments** |
| --- | --- |
| Dana-Farber Boston Children’s Cancer and Blood Disorders Center | 15 |
| Texas Children’s Hospital | 4 |
| Children’s Hospital of Philadelphia | 4 |
| UCSF Benioff Children’s Hospital | 3 |
| Total | 26* |

*Note: one patient became ineligible following enrollment prior to initiation of study treatment as noted in **Figure 1**.

**Supplemental Table 2**: Description of dose limiting toxicities in pediatric patients treated with fimepinostat (n=5 patients reported DLTs).

| **Age at enrollment (yrs)** | **Sex** | **Diagnosis** | **Number of Prior Treatment Regimens** | **Cycle** | **Toxicity Category CTCAE v4.0** | **Dose limiting toxicity description** | **Dose Level** |
| --- | --- | --- | --- | --- | --- | --- | --- |
| 18.4 | Female | Alveolar Rhabdomyosarcoma | 3 | 1 | Gastrointestinal disorders | Grade 3 Diarrhea | Dose Level 3 |
| 4.1 | Female | Neuroblastoma | 1 |  | Investigations | Grade 4 Neutrophil count decreased | Dose Level 2 |
| 6.5 | Female | Neuroblastoma | 8 |  |  | Grade 4 Platelet count decreased | Dose Level 3 |
| 11.0 | Male | Osteosarcoma | 6 |  | Metabolism and nutrition disorders | Grade 3 Acidosis | Dose Level 3 |
| 19.6 | Female | Myxoid Liposarcoma | 3 |  | Nervous system disorders | Grade 3 Headache | Dose Level 3 |
| 11.0 | Male | Osteosarcoma | 6 | 2 | Investigations | Grade 4 Neutrophil count decreased | Dose Level 3 |
| 4.1 | Female | Neuroblastoma | 1 | 3 | Investigations | Grade 4 Neutrophil count decreased | Dose Level 2 |
